# Exchange scaling of ultrafast angular momentum transfer in 4*f* antiferromagnets

**DOI:** 10.1038/s41563-022-01206-4

**Published:** 2022-02-24

**Authors:** Y. W. Windsor, S.-E. Lee, D. Zahn, V. Borisov, D. Thonig, K. Kliemt, A. Ernst, C. Schüßler-Langeheine, N. Pontius, U. Staub, C. Krellner, D. V. Vyalikh, O. Eriksson, L. Rettig

**Affiliations:** 1 Department of Physical Chemistry, Fritz Haber Institute of the Max Planck Society, Berlin, Germany; 2 Department of Physics and Astronomy, Uppsala University, Uppsala, Sweden; 3 School of Science and Technology, Örebro University, Örebro, Sweden; 4 Physikalisches Institut, Goethe-Universität Frankfurt, Frankfurt am Main, Germany; 5 Institute for Theoretical Physics, Johannes Kepler University, Linz, Austria; 6 Max-Planck-Institut für Mikrostrukturphysik, Halle (Saale), Germany; 7 Helmholtz-Zentrum Berlin für Materialien und Energie, Berlin, Germany; 8 Swiss Light Source, Paul Scherrer Institut, Villigen, Switzerland; 9 Donostia International Physics Center (DIPC), Basque Country, Spain; 10 IKERBASQUE, Basque Foundation for Science, Bilbao, Spain

**Keywords:** Ultrafast photonics, Magnetic properties and materials

## Abstract

Ultrafast manipulation of magnetism bears great potential for future information technologies. While demagnetization in ferromagnets is governed by the dissipation of angular momentum^1–3^, materials with multiple spin sublattices, for example antiferromagnets, can allow direct angular momentum transfer between opposing spins, promising faster functionality. In lanthanides, 4*f* magnetic exchange is mediated indirectly through the conduction electrons^4^ (the Ruderman–Kittel–Kasuya–Yosida (RKKY) interaction), and the effect of such conditions on direct spin transfer processes is largely unexplored. Here, we investigate ultrafast magnetization dynamics in 4*f* antiferromagnets and systematically vary the 4*f* occupation, thereby altering the magnitude of the RKKY coupling energy. By combining time-resolved soft X-ray diffraction with ab initio calculations, we find that the rate of direct transfer between opposing moments is directly determined by this coupling. Given the high sensitivity of RKKY to the conduction electrons, our results offer a useful approach for fine tuning the speed of magnetic devices.

## Abstract (web summary)

By exploring ultrafast magnetization in several compounds with similar crystal structures but different 4*f* magnetic elements, the authors show that the Ruderman–Kittel–Kasuya–Yosida interaction controls the spin dynamics.

## Main

Lanthanides are increasingly important in technology because their 4*f* spin moments reach exceptionally large sizes compared to those of 3*d* transition metals. For applications involving ultrafast spin dynamics, however, the localized nature of 4*f* magnetism poses an additional challenge compared to its 3*d* counterpart. Highly confined to the space near their ion, the magnetic 4*f* electronic states generally are not conduction electrons as in the 3*d* case, but lie several electronvolts below the Fermi level. Therefore, 4*f* electrons are typically not directly optically excited. Instead, optical pulses excite the conduction electrons, which mediate the RKKY coupling between the 4*f* spins. In equilibrium, RKKY acts as a Heisenberg exchange, with a coupling energy expressed as^5^ *j* ∝ |*I*|^2^*χ*, in which *χ* is the non-local susceptibility of the conduction electrons and *I* is the on-site exchange integral between the 4*f* states and the conduction electrons^6^. The participation of dispersive electronic states renders RKKY exceptionally sensitive to external factors. For example, the oscillating nature of *χ* is central for the large variation in magnetic ground states in the lanthanide metals^5^. *χ* also promises new routes towards ultrafast control of the RKKY coupling between 4*f* moments, such as by tuning the electronic occupation near the Fermi level. Similarly, the strength of the RKKY interaction depends strongly on the on-site exchange integral *I*, which is determined by the orbital overlap of the 4*f* and conduction electron clouds, and therefore strongly depends on the 4*f* occupation.

This warrants a systematic investigation into the role of 4*f* occupation on ultrafast magnetization dynamics. Previous attempts to address this question have focussed on ferromagnetic (FM) lanthanide metals^7^, which limits the comparison to three heavier lanthanides (Gd, Tb and Dy) and rules out demagnetization channels that do not involve interactions with the crystal lattice, such as the transfer of angular momentum between antiparallel spins. While reports of ultrafast 4*f* spin dynamics in antiferromagnets are scarce^8–10^, one experiment performed on an antiferromagnetic (AF) lanthanide suggested the existence of this channel, which has been proposed as a route to overcome speed bottlenecks associated with the lattice^9^. However, a systematic study of elemental lanthanide metals is hindered by the large variety of different crystal structures and magnetic phases they exhibit, such as spin helixes and spin spirals^5^, further complicating a meaningful comparison.

Here, we facilitate a direct comparison of 4*f* dynamics under comparable conditions by studying ultrafast magnetization dynamics in a series of lanthanide intermetallic antiferromagnets with nearly identical crystal and magnetic structures all across the lanthanide series. This approach allows us to single out the influence of 4*f* occupation. While demagnetization timescales are found to differ by nearly two orders of magnitude between materials, the corresponding angular momentum transfer rates clearly exhibit a scaling relation known as de Gennes scaling. Our ab initio calculations identify this as transfer between antiparallel moments and show that it scales with the magnitude of the RKKY coupling between them. Our approach provides a microscopic picture of such AF angular momentum transfer, yielding insight substantially beyond phenomenological models^11–13^, which often do not consider this transfer channel.

**Fig. 1:**
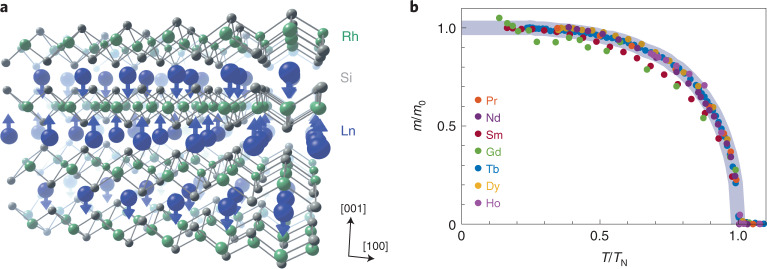
The LnRh_2_Si_2_ materials. **a**, The layered crystal structure, highlighting the layer-by-layer antiferromagnetic reversal along the [001] direction (for Ln = Sm and Gd, moments lie in-plane). **b**, Temperature dependence of the Ln^3+^ sites’ ordered 4*f* moment in all materials, exhibiting mean-field-like behaviour. The axes are normalized by each material’s saturated moment *m*_0_ and Néel temperature *T*_N_. The data were extracted from temperature-dependent resonant magnetic X-ray diffraction experiments (Methods). The grey line is a guide for the eye representing mean-field behaviour.

We consider a series of antiferromagnets of the form LnRh_2_Si_2_ (Ln is a lanthanide element). These share the same crystal structure and 4*f* spin arrangement (with <2.5% change in lattice parameters; Supplementary Section 3), such that the only appreciable difference between them is the occupation of the Ln ions’ 4*f* shell. Their collinear, compensated AF arrangement (Fig. 1a) exhibits a mean-field-like temperature dependence (Fig. 1b) and removes the need for considering stray fields and domain effects (Supplementary Section 1.2), which are necessary when considering ferromagnets. As such, these materials can be regarded as a lattice of AF-ordered Ln ions in a Rh_2_Si_2_ cage and can serve as an ideal test bed for comparing dynamics of the 4*f* moments with varying 4*f* occupation.

**Fig. 2:**
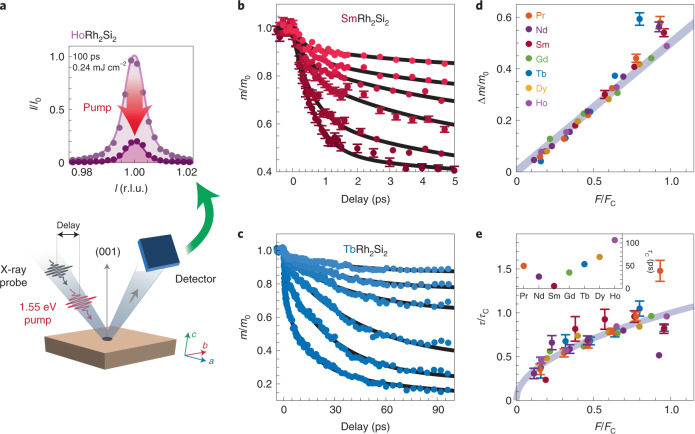
The 4*f* magnetization dynamics probed in diffraction. **a**, Sketch of the experimental scheme, with the scattering vector parallel to the sample’s [001] crystal direction and the two pulses arriving collinearly. The graph on top shows reciprocal space scans of the (001) magnetic reflection before excitation (bright) and 100 ps after excitation (dark) using an absorbed fluence of *F* = 0.24 mJ cm^−2^ (*F*/*F*_C_ = 2.6). r.l.u. are reciprocal lattice units. **b**,**c**, Pump-induced changes in the antiferromagnetically ordered 4*f* moment for Ln = Sm and Tb, respectively, representing data from both light and heavy lanthanides, highlighting the large difference in timescales and fluences. Different curves (in differently shaded color) correspond to different pump fluences: from *F*/*F*_C_ = 0.38 to 1.9 for Sm (0.05 to 0.28 mJ cm^−2^) and from *F*/*F*_C_ = 0.32 to 3.2 for Tb (0.26 to 2.6 mJ cm^−2^). **d**, Total demagnetization amplitude as a function of normalized fluence for all materials (Methods; line is a guide for the eye). **e**, Exponential time constant of the dominant (slower) drop as a function of normalized fluence. The data are normalized to *τ*_C_, the value at *F*_C_ (inset). *τ*_C_ values are extracted for each compound from the best fit between all shown data and the relation $\tau /\tau _{\mathrm{C}} = \sqrt {F/F_{\mathrm{C}}}$ (grey curve; Supplementary Section 5). Errors are defined in the Methods section.

We study this AF order using resonant magnetic soft X-ray diffraction. Exclusive sensitivity to the 4*f* moments is achieved by tuning the incoming photon energies to the Ln ions’ M_4,5_ resonances (3*d* → 4*f* excitations). The AF-ordered moment *m* is extracted from the intensity of the magnetic Bragg reflection (normalized to its saturated value *m*_0_; Fig. 2a and Supplementary Section 1). To achieve the high temporal resolution needed for this experiment, we used ultrashort X-ray pulses produced by the femto-slicing facility ‘FemtoSpeX’ at BESSY II (ref. ^14^).

We excite the materials with 1.55 eV laser pulses, and the response is qualitatively identical in all materials: the excitation suppresses the ordered AF moment in a process that begins with a fast (subpicosecond) drop, followed by a second slower drop (Fig. 2b,c). The fast drop accounts for a smaller fraction of the total reduction (except for Ln = Sm), and is vanishingly small for the heaviest Ln ions studied (Dy and Ho). However, quantitatively the materials’ response times vary widely, ranging from ~1 ps to over 100 ps.

For systematically comparing the behaviour we observe in the LnRh_2_Si_2_ family, the excitation fluence was varied. The total reduction in *m* scales linearly with fluence up to a material-dependent critical fluence *F*_C_, which also varies widely between materials. We define *F*_C_ as the fluence at which the total demagnetization amplitude Δ*m* reaches *m*_0_/2. Figures 2d,e present the total demagnetization amplitude *m*/*m*_0_ and the dominant (slower) time constant. The data are presented as functions of normalized fluence *F*/*F*_C_, and the time constants *τ* are also normalized by *τ*_C_, their values at the critical fluence *F*_C_ (inset), demonstrating similar scaling in all materials, despite the markedly different timescales and 4*f* filling. Exact *τ*_C_ values are extracted by fitting all data in Fig. 2e for each material (Methods).

**Fig. 3:**
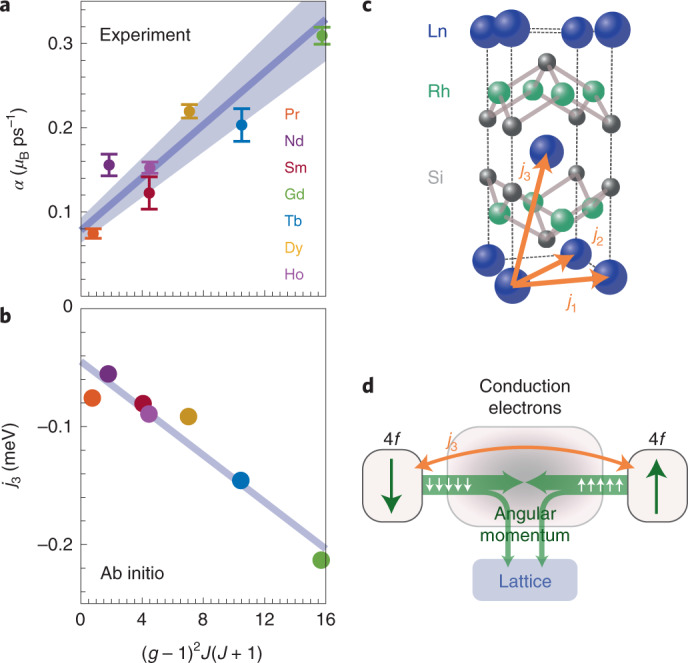
De Gennes scaling and RKKY coupling. **a**, Experimental values of the maximal angular momentum transfer rates (explanation in main text) as a function of the de Gennes factor *G*. Data are shown for *F*/*F*_C_ = 0.37 (other fluences behave very similarly). The best fit to a linear trend is presented, with a shaded area representing the error margin (slope: (15 ± 2)10^−3^ *μ*_B_ ps^−1^; offset: (78 ± 14 )10^−3^ *μ*_B_ ps^−1^). **b**, Calculated RKKY coupling between the nearest antiferromagnetically aligned Ln ions, also plotted against *G*. The line is a guide for the eye. **c**, Sketch of an extended unit cell with the nearest RKKY couplings indicated; *j*_3_ is the interlayer coupling. **d**, Diagram depicting the flow of 4*f* angular momentum after excitation, in which conduction electrons mediate the flow between 4*f* states on antiparallel sites, as well as the flow to the lattice. Errors are defined in the Methods section.

Two-step demagnetization is typical for lanthanide systems^11^.The two timescales are understood as one process, which slows down when thermalization of the electronic and lattice degrees of freedom occurs before demagnetization is complete^11,15^. Such a case is expected for the large 4*f* moments of many lanthanides, which require more time to release their angular momentum, compared to the smaller moments in transition metal 3*d* systems. Nevertheless, different Ln^3+^ ions vary appreciably in their moment sizes *μ*_B_*gJ* (*μ*_B_, *g* and *J* are Bohr’s magneton, the Landé factor and the total 4*f* angular momentum quantum number, respectively), ranging from 0.7*μ*_B_ to 10*μ*_B_. To account for the varying moment sizes, and given the universal dynamics observed in Fig. 2, we facilitate a more direct comparison of the demagnetizations by considering angular momentum transfer rates *α*, in units of *μ*_B_ ps^–1^ (exact definition in the Methods). These are calculated separately for the two demagnetization steps from the total moment *J* (Methods), but since they both represent the same physical process, we focus on the slow step (Fig. 3a), which we clearly resolve in all compounds. We find that *α* exhibits a linear relation to the de Gennes factor *G* = (*g* – 1)^2^*J*(*J* + 1), which approximates the projection of the spin **S** on **J**, squared^16^. De Gennes scaling has been experimentally demonstrated in several 4*f* systems^16,17^ for quantities including the interlayer spin turn angle^17,18^ and magnetic ordering temperatures^19^, and therefore also for the strength of RKKY coupling^16^.

The linear relation we observe strongly suggests that ultrafast demagnetization in LnRh_2_Si_2_ antiferromagnets depends on the strength of RKKY coupling between antiferromagnetically aligned moments and is therefore governed by the angular momentum transfer between opposite spins. To test this, ab initio calculations of all primary RKKY couplings were performed. These predict that the interplanar coupling *j*_3_ (between antiparallel spins) indeed scales linearly with *G* (Fig. 3b). By contrast, the in-plane couplings *j*_1_ and *j*_2_ (Fig. 3c) do not show a clear trend with *G* (Supplementary Section 7).

The linear scaling in Fig. 3a does not cross the origin. This suggests a contribution from an additional angular momentum transfer channel, independent of *G*, and therefore independent of 4*f* occupancy (that is, a process that is nearly the same in all LnRh_2_Si_2_ materials). One such process is the dissipation of 4*f* angular momentum to the lattice through the conduction electrons. To analyse this, one can consider a scenario in which angular momentum transfer between opposing 4*f* spins is turned off. The 4*f* demagnetization would then depend on two processes, (1) the transfer of 4*f* angular momentum to the conduction electrons, and (2) its dissipation from there to the lattice. The first process is governed by on-site exchange (and therefore by *G*), so one could assume that it is faster than the second process. However, since the conduction electron moment is small, it represents a bottleneck for angular momentum transfer such that process (1) is limited by the rate of process (2), and the observed 4*f* demagnetization would thus be limited by process (2) in a similar way in all LnRh_2_Si_2_ materials. When the 4*f* spin–spin channel is turned back on, it works in parallel to process (2), so this limit is relaxed by the additional angular momentum transfer rate, leading to the linear trend in Fig. 3a. The angular momentum transfer scenario we describe is sketched in Fig. 3d. The bottleneck aspect is similar to the case of *sp–d* FM semiconductors^20,21^. Previous works have discussed another channel primarily in the context of FM systems, in which the 4*f* shell couples directly to the lattice^7,22^. Such a channel should depend on the 4*f* occupation and on *G* via the strength of spin–orbit coupling, which shows a non-monotonous dependence on *G* (ref. ^5^). While our data confirm that the dominating contribution to the angular momentum transfer rates depends on the strength of the RKKY interaction, we cannot rule out additional contributions within the scatter of the data around the line in Fig. 3a.

Our results underline the importance of angular momentum transfer directly between opposite moments, as a channel that can dominate the entire process. This is in line with reports in other RKKY-mediated systems, such as the AF phases of lanthanide metals. Notably, in metallic Dy, which harbours FM and AF phases in different temperature ranges, an efficient demagnetization channel in the AF phase was recently observed, which is absent in the FM phase^9^. This is understood as the RKKY-mediated spin–spin channel we discuss here, and these observations are also in line with 4*f* demagnetization in AF metallic Ho^8^. However, demagnetization in the FM systems Tb and Gd reportedly also exhibited an ultrafast channel^7^ like AF Dy. The authors of the Dy work^9^ concluded that this was an extrinsic effect due to spin transport. The differences in demagnetization rates between these three isostructural ferromagnets (Gd, Tb and Dy) were therefore understood as due to different coupling strengths between the 4*f* shell and the lattice, with a particularly weak coupling for the half-filled 4*f* shell of Gd.

In conclusion, we have investigated the role of direct angular momentum transfer between spin sublattices in the ultrafast magnetization dynamics of 4*f* antiferromagnets. By a systematic comparison of the ultrafast angular momentum transfer rates with ab initio calculations, we find that the rate of this transfer channel is proportional to the magnitude of the antiferromagnetic indirect (RKKY) exchange coupling. Our findings open avenues for ultrafast control of magnetization, for example, by tuning indirect exchange coupling through manipulation of the conduction electrons via doping, voltage biasing or applied pressure, or even transiently, for example through photodoping, without affecting the magnitude of the 4*f* moments themselves. The implications of our results are not limited purely to antiferromagnets, as direct angular momentum transfer can also occur between inequivalent spins in, for example, ferrimagnets^23–25^ or alloys^26^ such as Gd_1–*x*_Tb_*x*_, where direct Gd–Tb angular momentum transfer was demonstrated^27^. Such control over angular momentum transfer rates is also essential for the design and optimization of new device functionalities, such as ultrafast all-optical switching, which has been shown to depend on angular momentum transfer between magnetic sublattices^28,29^. The ability to tune the demagnetization rate of selected sublattices and the transfer rate between them opens the possibility to engineer such devices, either shortening or prolonging the short-lived collective spin states that enable such effects^30^.

## Methods

### Sample preparation

Samples were single crystals of all seven materials (LnRh_2_Si_2_ with Ln = Pr, Nd, Sm, Gd, Tb, Dy, Ho), grown as previously described^31^. Due to the layered crystal structure, the sample surface is precisely perpendicular to the tetragonal [001] axis. The crystals used were approximately 1–2 mm^3^ in size, with faces much larger than the pump and probe beam spots. Néel temperatures found in our experiments agree with literature values^31^: for Ln = Pr, Nd, Sm, Gd, Tb, Dy and Ho, *T*_N_ = 68 K, 58 K, 64 K, 107 K, 94 K, 52 K and 29 K, respectively.

### Resonant X-ray diffraction

All experiments were conducted by fulfilling the Bragg condition for the (001) magnetic reflection using incoming photon energies near the Ln ion’s respective dominant M edge: M_4_ for Pr, Nd and Sm (3*d*_3/2_ → 4*f*); and M_5_ for Gd, Tb, Dy and Ho (3*d*_5/2_ → 4*f*). The spectral shapes of the edges are shown in Supplementary Fig. 1. From the width of the (001) reflection around these edges, we estimate effective probe depths of ~4 nm for Ln = Gd and Sm, ~5 nm for Tb and ~7 nm for Pr, Nd, Dy and Ho. All experiments were conducted with *σ*-polarized incoming X-ray light, such that only the *σ* → *π*′ scattering channel contributes to the magnetic signal. For all materials except GdRh_2_Si_2_, the observed dynamics and temperature dependences do not depend on the azimuthal orientation of the sample around the surface normal, so that the ordered moment *m* in Figs. 1 and 2 is extracted from diffracted intensity as $m \propto \sqrt I$ (Supplementary Section 3). The procedure for extracting *m* from GdRh_2_Si_2_ is detailed elsewhere^10^.

Equilibrium resonant X-ray diffraction experiments were conducted at beamline X11MA of the Swiss Light Source^32^ using the RESOXS end station^33^, and at the PM3 beamline at the Helmholtz-Zentrum Berlin. Time-resolved resonant X-ray diffraction experiments were conducted in a ultrahigh vacuum scattering chamber using ultrashort X-ray pulses from the femto-slicing facility at beamline UE56/1-ZPM (ref. ^14^) at Helmholtz-Zentrum Berlin. The zone plate monochromators used in this experiment provide an energy resolution typically of ~5 eV (Supplementary Fig. 1). Data for GdRh_2_Si_2_ is taken from a previously reported experiment^10^.

The pump–probe experimental scheme was conducted at 3 kHz using 1.55 eV (800 nm) *p*-polarized pump pulses. The X-ray repetition rate is 6 kHz such that between every pumped event an unpumped signal is recorded, and no average heating was observed for the presented data. X-ray intensities were collected using an avalanche photodiode, in a scheme allowing for single-photon counting. As such, the error bars in Fig. 2a–c are taken as ${{{\mathrm{{\Delta}}}}}I = \sqrt I$. Reciprocal space scans (as in Fig. 2a) showed no peak broadening or shifting, so only the peak heights were collected in time traces. The X-ray and 1.55 eV pulses arrive nearly collinearly, but the avalanche photodiode does not collect the pump photons, as they are filtered by an Al foil. The X-ray spot size was always smaller than the pump spot but inevitably varied between experiments on different samples because the experiments were conducted in the Helmholtz-Zentrum Berlin user facility over several years. To overcome this, at the beginning of every experiment, a sample from the previous experiment was remeasured. The laser excitation was then adjusted to ensure that the exact same fluence-dependent response was observed as in the previous experiment. Exact values for each experiment are available in Supplementary Section 2.

All demagnetization curves, such as in Fig. 2b,c, were fit to an equation of the form

1 $$\frac{{m\left( t \right)}}{{m_0}} = 1 - {{{\mathrm{{\varTheta}}}}}\left( t \right)\left( {d_{{\mathrm{fast}}}\left( {1 - {\mathrm{e}}^{ - t/\tau _{{\mathrm{fast}}}}} \right) + d_{{\mathrm{slow}}}\left( {1 - {\mathrm{e}}^{ - t/\tau _{{\mathrm{slow}}}}} \right)} \right),$$

in which *Θ* is the Heaviside function, *d*_slow/fast_ is the demagnetization amplitude, and *τ*_slow/fast_ is the exponential time constant associated with the slow/fast process. The temporal resolution is estimated at 120 fs. To account for this, fits to equation (1) were conducted with a convolved Gaussian response function of 120 fs width. For the case of Ln = Dy and Ho, a subpicosecond contribution was not observed, so *d*_fast_ was set to 0. Error bars in Figs. 2d,e and 3a represent standard deviations of these fit results.

The critical fluence *F*_C_ is defined for each material as the fluence at which the total observed reduction in *m*(*t*)/*m*_0_ (that is, *d*_fast_ + *d*_slow_) reaches an amplitude of 0.5. The values of *F*_C_ are found to scale with *T*_N_*S*^–1^, such that *F*_C_ may serve as a measure of an effective transient Weiss field (Supplementary Section 4). Timescales are expected to scale with the square root of such fields (Supplementary Section 5), so the *τ*_C_ values in the inset of Fig. 2e are calculated as the best fit to $\tau _{{\mathrm{slow}}}/\tau _{\mathrm{C}} = \sqrt {F/F_{\mathrm{C}}}$.

The maximal angular momentum transfer rate is calculated as *α*_*x*_ = *m*_0_*d*_*x*_*τ*_*x*_^–1^, in which *x* represents ‘fast’ or ‘slow’ from equation (1); *m*_0_ is the ordered 4*f* moment, which is taken as the theoretical value of *gμ*_B_*J* (*g* is the Lande’ factor, *μ*_B_ is Bohr’s magneton and *J* is the total angular momentum) and adjusted according to Fig. 1b, to account for the finite initial temperature.

### Ab initio calculations

Two independent calculations were done to confirm the theoretical trend in *j*_3_. The exchange coupling parameters presented in Fig. 3b were calculated using a self-consistent Green’s function method^34,35^ within density functional theory in a generalized gradient approximation (GGA)^36^. Strongly localized 4*f* electrons were treated within the GGA + *U* approach^37^. The corresponding effective Hubbard parameter U* = *U* − *J* was chosen in such a way as to guarantee a good agreement of calculated and experimental Néel temperature. The exchange parameters were estimated using the magnetic force theorem implemented within the multiple scattering theory^38^. For Ln = Sm, Gd and Dy, the free *z* parameter of the Si ions’ position has not been reported, and the nominal value of 0.375 has been used.

In a second independent calculation, the electronic properties of LnRh_2_Si_2_ compounds were also calculated using density functional theory, as implemented in the all-electron full-potential fully relativistic electronic structure code RSPt (refs. ^39–41^) that uses linear muffin-tin orbitals as basis functions. This calculation also confirmed the linear trend of *j*_3_ presented in Fig. 3b. Details of this calculation and a comparison between the two calculations are available in Supplementary Sections 7 and 8.

To confirm the validity of these results, the total energy from the corresponding spin-Hamiltonian was minimized using a Monte Carlo calculation implemented in the simulation package UppASD (ref. ^13^). At zero temperature, this produced the experimentally observed ground states with in-plane ferromagnetic arrangements and out-of-plane antiferromagnetic arrangements of the magnetic moments.

Our calculations and analysis do not account for the point-ion contribution to the magnetocrystalline anisotropy energy (MAE), due to the complexity and ambiguity of its calculation from first-principles theory, in particular for lanthanide elements and compounds. This contribution is not expected to affect the results of the theoretical calculations nor to influence the conclusions of this work, because in lanthanides in general, the MAE is small compared to the exchange fields. This can be estimated, for example, from the lanthanide elements, where the crystal field parameters (which determine the MAE) are on the order of 100 μeV or lower^5^, which can be compared to the substantially larger exchange parameters calculated here (Supplementary Section 7 for details). Hence, point-ion-driven MAE is not expected to play a major role in determining the AF demagnetization processes discussed above.

## Online content

Any methods, additional references, Nature Research reporting summaries, source data, extended data, supplementary information, acknowledgements, peer review information; details of author contributions and competing interests; and statements of data and code availability are available at 10.1038/s41563-022-01206-4.

## Supplementary information

Supplementary Information Supplementary Figs S1–S7, Tables 1 and 2 and Sections 1–10.

## Data Availability

All datasets contributing to the results in this work are available on an online repository (10.5281/zenodo.5828162), including data collected upon equilibrium heating (Fig. 1b) and upon photoexcitation.
