# Supplementary material for: Exchange scaling of ultrafast angular momentum transfer in 4f antiferromagnets
Source: Nat Mater. 2022 Feb 24;21(5):514–7. doi: 10.1038/s41563-022-01206-4 (PMC9064787; doi:10.1038/s41563-022-01206-4)
Supplement: Supplementary file 1 — Supplementary Figs S1–S7, Tables 1 and 2 and Sections 1–10. [file 41563_2022_1206_MOESM1_ESM.pdf]

nature portfolio

https://doi.org/10.1038/s41563-022-01206-4

Supplementary information

Exchange scaling of ultrafast angular momentum transfer in 4*f* antiferromagnets

In the format provided by the authors and unedited

Supplementary material

Exchange scaling of ultrafast angular momentum transfer in 4*f* antiferromagnets

Y. W. Windsor[1], S-E. Lee[1], D. Zahn[1], V. Borisov[2], D. Thonig[3], K. Kliemt[4], A. Ernst[5,6], C. Schüßler-Langeheine[7], N. Pontius[7], U. Staub[8], C. Krellner[4], D. V. Vyalikh[9,10], O. Eriksson[2], L. Rettig[1]

[1] Department of Physical Chemistry, Fritz Haber Institute of the Max Planck Society, Faradayweg 4-6, 14195 Berlin, Germany
[2] Department of Physics and Astronomy, Uppsala University, Box 516, SE-75120 Uppsala, Sweden
[3] School of Science and Technology, Örebro University, SE- 70182 Örebro, Sweden
[4] Physikalisches Institut, Goethe-Universität Frankfurt, 60438 Frankfurt am Main, Germany
[5] Institute for Theoretical Physics, Johannes Kepler University, Altenberger Strasse 69, 4040 Linz, Austria
[6] Max-Planck-Institut für Mikrostrukturphysik, Weinberg 2, 06120 Halle (Saale), Germany
[7] Helmholtz-Zentrum Berlin für Materialien und Energie GmbH, Albert-Einstein-Str. 15, 12489 Berlin, Germany
[8] Swiss Light Source, Paul Scherrer Institut, 5232 Villigen PSI, Switzerland
[9] Donostia International Physics Center (DIPC), 20018 Donostia/San Sebastián, Basque Country, Spain
[10] IKERBASQUE, Basque Foundation for Science, 48013, Bilbao, Spain

Contents

1. Resonant X-ray Diffraction experiments (RXD) 2
   1.1 Extraction of changes in local moment 2
   1.2 Domain effects 2
   1.3 Spectroscopic features of the resonance 3
2. Excitation using 800 nm laser light 5
3. Validity of comparison between *Ln*Rh_2Si_2 materials 6
   3.1 Crystal structure 6
   3.2 Magnetic order 6
   3.3 Equivalence of time-resolved experiments 7
4. Material-dependent critical fluences 8
5. Fluence dependence of demagnetization time scales 9
6. Estimating Antiferromagnetic Demagnetization Rates 9
7. Calculated exchange couplings 11
8. The scalar-relativistic approximation in calculating electronic and magnetic structures 12
9. Fits to demagnetization data 14
10. Non-normalized parameters 15
11. References 16

1. Resonant X-ray Diffraction experiments (RXD)

Here we provide additional details about the resonant magnetic diffraction experiments[1]. The purpose of this experiment is to probe the long-range antiferromagnetic order by elastic scattering at an angle that fulfills Bragg's law for this order. In the present case, we probe magnetic order with a modulation vector defined by Miller indices (001), i.e. $\boldsymbol{Q} = \boldsymbol{c}^*$.

The cross section for magnetic scattering of x-rays is very small compared to charge scattering[2]. Therefore, the second major aspect of this technique is the use of specific photon energies that correspond to atomic resonances of a specific ion. The scattering cross section can then be resonantly enhanced by several orders of magnitude[3], such that the signal is effectively dominated by scattering from the resonant ions. In the present case, we use energies that correspond to the $M_{4,5}$ edges of lanthanides, where the scattering length can reach $200r_0$[3,4] (r_0 is the classical electron radius). The dominant resonant process is assumed to be an E1 electric dipole transition ($3d \rightarrow 4f$), meaning that the resonant magnetic signal we collect is primarily sensitive to the 4f states.

1.1 Extraction of changes in local moment

For an electric dipole-dipole transitions (E1-E1 event), the intensity of a magnetic reflection I probed by RXD is proportional to the squared structure factor F, written as[3,5]

$$F(\mathbf{Q}) = (\hat{\boldsymbol{\varepsilon}}' \times \hat{\boldsymbol{\varepsilon}}) \cdot \sum_i \boldsymbol{f}_i e^{i\mathbf{Q}\cdot\mathbf{r}_i} \propto (\hat{\boldsymbol{\varepsilon}}' \times \hat{\boldsymbol{\varepsilon}}) \cdot \boldsymbol{m} \tag{S1}$$

Here the $(\hat{\boldsymbol{\varepsilon}}' \times \hat{\boldsymbol{\varepsilon}})$ term is the cross product between the incoming and scattered polarization vectors, $\boldsymbol{Q}$ is the scattering vector, and the sum is over all resonant ions in the magnetic unit cell (other ions are neglected, as their scattering contribution is non-resonant), each with position $\boldsymbol{r}_i$. The resonant scattering factor $\boldsymbol{f}_i$ is proportional to the local moment $\boldsymbol{m}$.

In the second step of Eq. (S1), we describe the present experiment by evaluating the sum for the magnetic (001) reflection, summing only over the resonant *Ln* ions. The sum in Eq. (S1) is then proportional to $\boldsymbol{m}$, such that the proportionality encodes the spectroscopic features of the resonance (see following), and the dot product represents the anisotropy of the scatterers (i.e., the magnetic moments) with respect to the scattering plane. Furthermore, this means that the respective intensity can be expressed as $I(x) \propto |\boldsymbol{F}|^2 \propto m^2(x)$, with x representing pump-probe time delay (t) or temperature (T).

This illustrates that for the case of $Ln\mathrm{Rh_2Si_2}$ materials, relative changes in the ordered 4f moment can be directly extracted from intensity. Exceptions to this are coherent rotations of all spins, resulting from changes to the anisotropy of the 4f system with T or t. This manifests through the dot product in Eq. (S1). In our experiments this occurs only in the case of *Ln* = Gd, for which the we employ a special procedure to correctly extract $m(x)$, as described in detail in a previous publication[6].

1.2 Domain effects

The presence of antiferromagnetic domains can affect the RXD signal if more than one domain is probed by the X-ray spot. The magnetic modulation in these domains is the same, though separated by a phase, complicating the analysis using Eq. S1. Here we consider the effect this could have on our experiments.

All experiments reported in this work were conducted with incoming σ polarized (linear) X-rays, so only the $\sigma \rightarrow \pi'$ polarization channel is considered because $\sigma \rightarrow \sigma'$ is zero by symmetry. This simplifies Eq. S1, and what remains is to consider the orientations of the moments in the unit cell. Two limiting cases exist:

1. **moments are alternatingly aligned along the $[001]$ axis**. This occurs for most of the studied materials (*Ln* = Pr, Nd, Tb, Dy, and Ho). In this case Eq. S1 yields $I \propto m^2$, meaning that the intensity is insensitive to antiferromagnetic domain effects that cause $m \to -m$. Furthermore, 90° twinning caused by the tetragonal symmetry of the crystals does not affect this signal either.
2. **alternating moments lie in the planes normal to $[001]$** (*Ln* = Gd and Sm). Here Eq. S1 yields $I \propto m^2 \cos^2 \Psi$, in which Ψ represents azimuthal rotation of the sample around $[00l]$. The signal remains insensitive to domain effects of the form $m \to -m$. Unlike the previous case, 90° twinning can affect the validity of using Eq. S1, because domains of $\propto \cos^2 \Psi$ and $\propto \cos^2(\Psi + 90°)$ can be simultaneously probed by our X-ray beam. This effect can influence our results if the temperature- or delay-dependent behavior of the system includes changes in the local magnetic anisotropy. This concern is relevant only to Ln = *Gd*, and our previous report[6] provides a detailed account of how this was overcome.

We therefore conclude that our experiments are insensitive to domain effects, except for the case of *Ln* = Gd, which was carefully accounted for.

1.3 Spectroscopic features of the resonance

In this section we consider how the behavior of the (001) reflection varies through the M edges used in our experiments. These resonances are the main difference between the experiments. Data were collected at beamlines with high energy resolution (either using the ReSoXS end station[7] at the SIM beamline[8] of the Swiss Light source, or at the PM3 beamline in HZB[9]), so that a reliable description of the resonant behavior of the (001) reflection is assumed in the data presented below.

In contrast, the time-resolved experiments were conducted at the FemtoSpeX facility at beamline UE56/1-ZPM[10], in which a zone plate monochromator (ZPM) is used to maximize photon flux. A property of the ZPM is its low energy resolution, such that a reliable description of the resonance is not possible.

At the high-resolution beamtimes, features of the atomic resonance are extracted by conducting reciprocal space scans through the (001) reflection ($\theta - 2\theta$ scans) at several energies around the absorption edge. This is presented in the left column of Fig. S1. The figure's rows each present data from a different *Ln* element. We focus on two quantities:

1. **Diffraction peak width (inverted)**, presented in the right-hand column as a function of energy, is a measure of the effective magnetic volume contributing to the diffraction signal. While off resonantly the width is dominated by the magnetic correlation length, at resonance it becomes limited by the finite penetration depth of the X-rays. We define the *effective probe depth* as half this value (light must travel in and out).
2. **Integrated intensity**, presented in the middle column as function of energy (solid icons). Equivalent energy scans were taken using the ZPM in the time-resolved experiment (open circles). To estimate the spectral resolution at each resonance, the high-resolution data were convolved with a Gaussian resolution function. This is presented as solid lines, and Gaussian width (at half maximum) is indicated in units of eV for each element. Values are listed in Table 1.

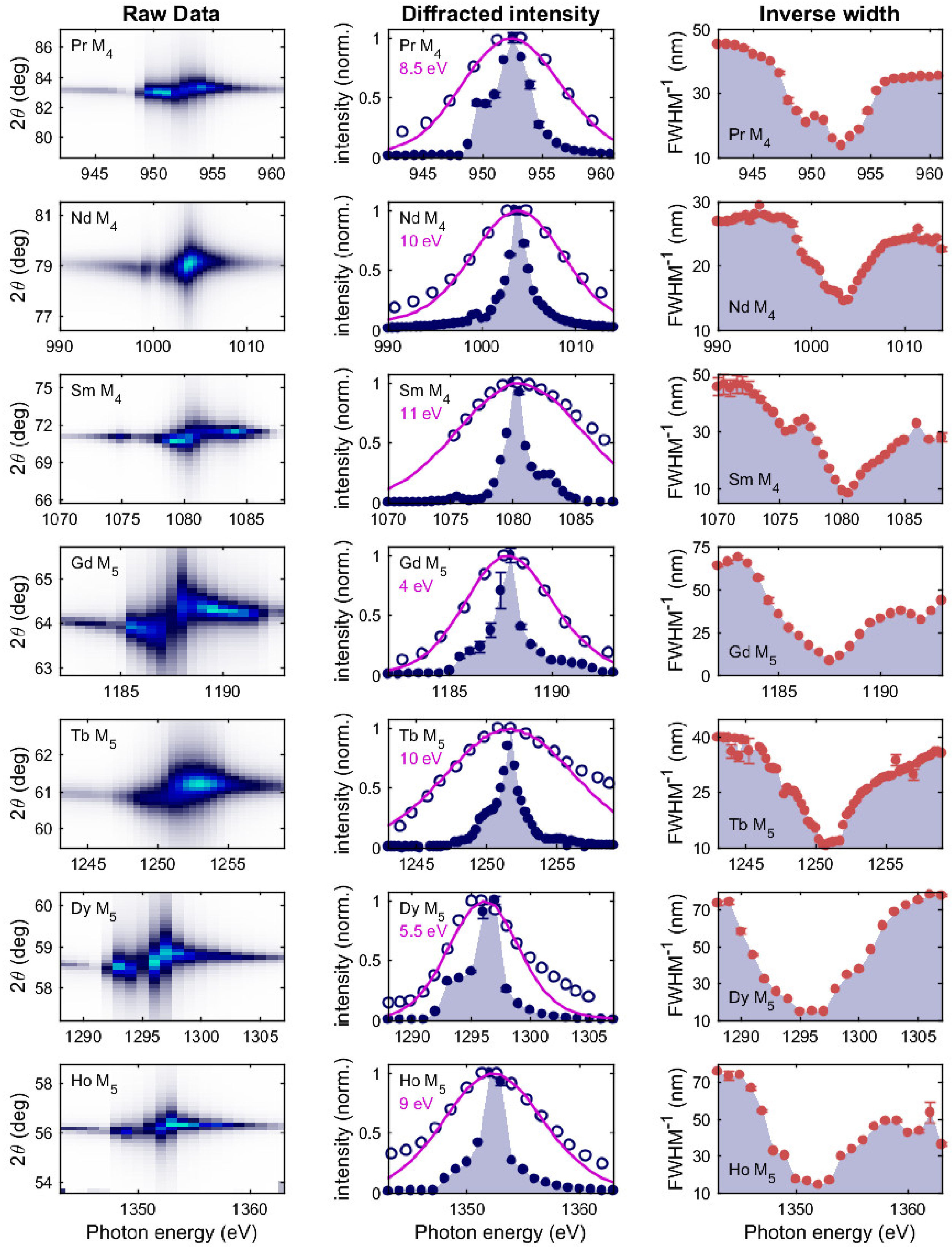

Fig. S1 – **The M edge resonances used in this experiment, probed through the (001) magnetic reflection.** The left column presents $\theta - 2\theta$ scans (equivalent to reciprocal space cuts along [001]) at several energies across the resonance. These scans were conducted at beamlines with high energy resolution. The color scale represents scattered intensity. The middle column presents the integrated intensity extracted from such scans (solid circles), alongside the energy scans of (001) taken at FemtoSpeX (open circles). The solid line is an estimate of the energy resolution at FemtoSpeX, produced by a convolution of the high-resolution data with a Gaussian of the indicated width in energy (full width at half maximum). The right column presents the inverse width of the Bragg reflections, providing a measure of the probe depth as function of energy.

2. Excitation using 800 nm laser light

The optical constants of the LnRh$_2$Si$_2$ materials are not available in literature. To assess the validity of comparing the experiments on these materials, we obtained the optical constants of all materials for $\lambda = 800\ nm$ light by measuring angle-dependent reflectivity. Using the Fresnel equations, we extracted the values as $n = n_0 + ik$.

A second aspect to consider is the incident angle of the pump light. In this experimental geometry, the pump light arrives nearly-collinear to the X-rays ($\sim 1°$ offset). The X-ray angle is defined by Bragg's law, and therefore varies between the resonances of the different materials (see Fig. S1, vertical axes in the left column). Using Snell's law, we estimate the refracted angles of the pump beam to the surface normal inside the material ϕ, and find that they are nearly identical in the range $15° - 18°$. Finally, the pump penetration depth is estimated as $\lambda(4\pi k)^{-1} \cos\phi$.

The pump spot size was estimated as the full-width at half maximum of the spot profile (see Table 1). The probe spot was in the vertical direction cut to 40 μm (except for GdRh$_2$Si$_2$, in which it was 170 ± 66 μm). In the horizontal direction the probe spot was kept to half the size of the pump spot.

Table 1 – effective values of relevant properties for the pump and probe beams (*note: for Gd and Sm the error of k is ± 0.2). The reflectivity of the pump R is calculated for the corresponding Bragg angle.

LnRh$_2$Si$_2$	n_0 (± 0.1)	k (± 0.1)	Pump depth (nm)	Pump spot ($\mu m \times \mu m$)	R	ZPM resolution (eV)	Probe depth (nm)
Pr	3.3	3.1	20	310 ± 6 × 345 ± 6	0.67	8.5	6.9
Nd	3.4	3.5	17	394 ± 6 × 433 ± 6	0.71	10.0	7.3
Sm	3.7	3.4	18	411 ± 5 × 545 ± 6	0.72	11.0	4.1
Gd	3.3	3.2	20	166 ± 26 × 321 ± 60	0.72	4.0	4.2
Tb	3.7	3.3	19	411 ± 5 × 545 ± 6	0.74	10.0	5.3
Dy	3.4	3.1	19	244 ± 36 × 241 ± 30	0.74	5.5	7.3
Ho	3.2	3.2	19	244 ± 36 × 241 ± 30	0.76	9.0	7.2

3. Validity of comparison between *Ln*Rh_2Si_2 materials

The validity of direct comparison between 4*f* dynamics of different *Ln*Rh_2Si_2 materials is central to this work. Here we discuss the validity of this comparison, and its limits.

3.1 Crystal structure

All studied materials crystallize in the tetragonal $ThCr_2Si_2$ structure, space group I4/mmm (#139). Their structures have been reported in various reports, most notably by Felner and Nowik[11,12], and more recently by Kliemt [13]. The main properties assumed in this work are listed in Table 1, and appear to vary very little. The lattice parameters change by <1.3 % to 2.2 %, leading to a ~4.5% maximal change in the unit cell volume, and ~3% change in the direct distance between nearest Ln ions in adjacent layers.

Table 2 – crystal structure parameters used in this work. a, c, V and z are the tetragonal lattice constants, unit cell volume and free parameter of the Si ions' position, respectively. The theoretical saturated 4*f* moment (*gJ*) and the distance between *Ln* ions in adjacent layers is also shown.

LnRh_2Si_2	a (Å)	c (Å)	V (Å^3)	z (r.l.u)	*gJ* (μ_B)	Ln-Ln (Å)	Ref.
Pr	4.079	10.14	168.68	0.3768	3.2	5.83	13,14
Nd	4.069	10.11	167.39	0.3725	3.27	5.82	13,15
Sm	4.055	10.04	165.09	/	0.71	5.78	13
Gd	4.042	9.986	163.15	/	7	5.75	13
Tb	4.037	9.95	162.16	0.3770	9	5.73	13,16
Dy	4.022	9.90	160.15	/	10	5.71	13
Ho	4.015	9.89	159.43	0.3765	10	5.70	13

3.2 Magnetic order

All investigated materials exhibit the same magnetic modulation of the 4*f* spins. The *Ln* layers in the *ab* plane are ferromagnetic, and adjacent layers are oppositely aligned, such that the ordering wave vector is (001). All measurements were conducted at temperatures corresponding to $T/T_N \leq 0.4$. In this temperature range magnetic excitations are nearly absent, as the specific heat capacity is nearly identical to that of isostructural non-magnetic $LuRh_2Si_2$ [13,17,18]. Two specific aspects requiring further clarification are:

- **Local ion anisotropy** – the arrangement of spins within the unit cell was determined through the azimuthal dependence of the (001) magnetic reflection (see methods section in the main text). All materials except for *Ln* = Gd and Sm exhibited no azimuthal dependence, indicating that the local anisotropy aligns the spins along the *c* axis (alternatingly parallel or antiparallel to it). For Sm and Gd the data confirmed that the 4f spins are aligned within the ab plane, while spins of adjacent planes remain antiparallel to each other. In the case of Gd the direction along which the spins point within the *ab* plane varies, as shown in Ref. [6]. The difference lays in the low anisotropy of the 4*f* shell in these ions compared to other *Ln* ions. This does not affect the on-site exchange coupling between the 4f electrons and the conduction electrons, so RKKY is not affected, justifying the systematic comparison between the materials' spin dynamics we performed. Furthermore, magnetic anisotropy energies in these systems are significantly smaller than the RKKY coupling energies.
- **Additional magnetic transitions** – two of the probed materials – *Ln* = Ho and Dy - exhibit an additional magnetic phase at very low temperatures, in which the 4*f* moments cant away from the (001)

direction. This phase is easily identifiable, because below its transition temperature, the intensity of the (001) magnetic reflection changes significantly. In both cases we conducted the experiments above this transition in the normal phase that is equivalent to the other materials in the *Ln*Rh_2Si_2 series in order to avoid this complication.

3.3 Equivalence of time-resolved experiments

Despite the great similarity between the materials, the different *Ln* ions require use of different *M* edge resonance energies for each material, rendering some differences between the experiments inevitable. Here we consider these differences.

The different *M* edge photon energies require different scattering angles to fulfill Bragg's law for the (001) reflection (see angles in the left column of Fig. S1). The effect that this has on the probe depth is already accounted for by the data in Table 1. However, since the pump arrives nearly collinearly with the probe, its angle also changes. This leads to only small differences in the refracted beam within the materials (15°-18°), and the reflection coefficient at the Bragg angle is also similar (around ~0.7). As such, we conclude that pump's depth profile is very similar in all materials.

The main concern surrounds the different probe depths, as shown in Table 1. These vary between the resonances by nearly a factor of two. This indicates that for different materials there is a different sensitivity to deeper layers. Deeper layers experience weaker pump excitation, reducing the observed pump effect. This would change the effective critical fluence values (see next section), and in worse case change the qualitative behavior observed. Since all materials still fall on the systematic scaling curves in the main text's Figures 2d, 2e and 3a, we conclude that this effect is minor. Furthermore, the critical fluences systematically scale with the Neel temperature (see next section), further indicating that effect of the varying probe depths is minor. Lastly, the variation in ZPM resolution (Table 1) also effects the probe depth, such that higher values increase the contribution of deeper layers. We find no qualitative effect of this issue.

4. Material-dependent critical fluences

The relations to the critical fluences shown in Figures 2d,e of the main text demonstrate a universal behavior with respect to the critical fluence F_C, which is different for each material. Here we show that the F_C values are intrinsic quantities reflecting the material-dependent energy scale required to observe the same qualitative behavior seen in all other materials.

Fig. S2 presents the F_C values extracted for each material as functions of T_N/S, the ratio between the Neel temperature and the theoretical 4f spin moment of the same material. This ratio is proportional to the Weiss molecular field, reflecting a measure of the mean magnetic coupling that is overcome by the excitation with F_C. Such a picture is valid when spin dynamics are dominated by classical processes, such that highly non-equilibrium processes or pure quantum fluctuations do not contribute.

We find that the optimal F_C values chosen for *Ln* = Sm and Pr do not scale as the rest of the materials. In the case of *Ln* = Sm, a significant portion of the total moment is demagnetized before electron-lattice thermalization (i.e. the sub-picosecond channel amplitude is larger), which can be attributed to its comparably smaller ordered 4f moments ($gJ = 5/7 \approx 0.7$). Quantum effects of such a small moment can be significant, and we propose that this is why $SmRh_2Si_2$ in Fig. S2 deviates from the linear behavior exhibited by most of the materials. The second outlier in Fig. S2 is $PrRh_2Si_2$. This system has in fact been previously studied, with focus on its unusually high ordering temperature of 70 K[19]. This is the reason for its deviation from the linear trend in Fig. S2, and a value closer to 35 K would place the value for $PrRh_2Si_2$ almost precisely on the interpolated curve. Shigeoka et al. demonstrated that the reason for this exceptionally large ordering temperature is the crystal-field-induced enlarged magnetic anisotropy, which causes this material to behave like an Ising magnet[19].

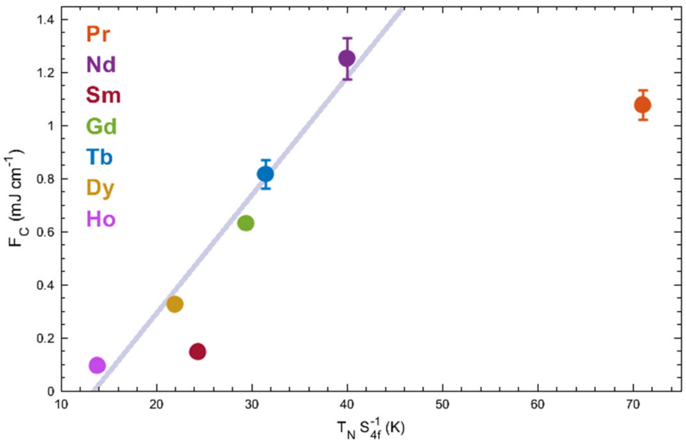

Fig. S2 – Critical absorbed fluence F_c as function of T_N/S, the ratio between the Neel temperature and the theoretical 4f spin moment.

5. Fluence dependence of demagnetization time scales

In this section we analyze the observed square root dependence of the exponential demagnetization time scales on the laser fluence (Fig. 2e in the main text). To this end, we interpret the underlying microscopic mechanisms in the demagnetization process, in the limit of low fluences. An effective spin temperature T is approximated to be linearly dependent on fluence F, which is the outcome of the empirical three-temperature model[20]. Temperature enters naturally into the equation of motion of the atomistic magnetic moments m_i at site i by the stochastic Landau-Lifshitz-Gilbert equation:

$$\frac{\partial \boldsymbol{m}_i}{\partial t} = \frac{-\gamma}{1+\alpha^2}\left(\boldsymbol{m}_i \times \boldsymbol{B}_i + \frac{\alpha}{m}\boldsymbol{m}_i \times (\boldsymbol{m}_i \times \boldsymbol{B}_i)\right). \tag{S2}$$

This describes the evolution of the moment as a superposition of precessional motion around, and dissipative motion towards an effective field $B_i = B_i^{ex} + b_i$. Here, the Weiss field $B_{ex} = -\frac{\partial H}{\partial m_i}$ is related to the underlying spin-Hamiltonian H. Furthermore, b is a thermal field with the constraints

$$\langle b_i(t)\rangle = 0$$
$$\left\langle b_i^{\mu}(t) b_j^{\nu}(t')\right\rangle = 2D\delta_{\mu\nu}\delta_{ij}\delta(t-t'), \tag{S3}$$

where $D = \alpha\frac{k_B T}{\gamma m}$ is the diffusion amplitude, α is the Gilbert damping, γ the gyromagnetic ratio, k_B is the Boltzmann constant, and m the moment's length. From the correlation of b_i it follows that the thermal field averaged over time scales as $\sqrt{T}$ and, consequently, also $\frac{\partial m_i}{\partial t}$. Upon excitation, the total magnetic moment $M = \frac{1}{N}\left|\sum_i m_i\right|$ demagnetizes exponentially (approximated as $M = M_0 e^{-\frac{t}{\tau}}$), with an amplitude M_0 that is linearly proportional to fluence[21]. Finally, we combine all relations with the fluence to reach:

$$\sqrt{F} \propto \frac{\partial M}{\partial t} = -\frac{1}{\tau}M \propto -\frac{M_0}{\tau} \propto -\frac{F}{\tau} \to \tau \propto \sqrt{F}. \tag{S4}$$

Dependences of demagnetization times on fluence have been previously observed experimentally and reproduced by calculations[22,23].

6. Estimating Antiferromagnetic Demagnetization Rates

For comparing between demagnetization rates calculated from first principles and experiments (Fig. 3a in the main text), we consider that demagnetization in the 4*f* antiferromagnets is governed by magnon processes; spin-mixing or optical inter-site spin transfer (OISTR) are suppressed due to the local character of the *f*-states. An estimate of magnon-induced magnetization dynamics can be obtained from the atomistic form of the Landau-Lifshitz-Gilbert (LLG) equation (S2) for the atomic moment $\vec{m_i} = m_i\vec{e_i}$ at site i as motivated above in Section 5.

It is important to note that not only the interlayer magnetic exchange, but all exchange interactions, including the RKKY couplings, enter the Weiss-field $\vec{B_i}$ via the Heisenberg model $H = -\sum J_{ij}\vec{e}_i\,\vec{e}_j$ **.**

Only two main quantities in the magnon-governed process are important for the de-, and remagnetization rates: the Weiss-field and the Gilbert damping[24]. To resolve their importance, we studied first the role of the Weiss-field in the demagnetization process of 4*f*-antiferromagnets, by putting the damping parameter (dissipation term) to zero. This assesses the hypothesis of Malik et al.[24], that the dissipation mechanism may be less dominant for the demagnetization process.

Using the LLG equation, assuming spin-spin interaction from the Heisenberg model, leads to a rough estimate of the relevant time scales

$$\left|\frac{\partial \vec{\boldsymbol{e}}_{\boldsymbol{i}}}{\partial t}\right| \approx \frac{\sum J_{ij}(\mathrm{meV})}{m(\mu_{\mathrm{B}})} \times 3.04 \times 10^{12}\ \mathrm{s}^{-1} \tag{S5}$$

where the sum includes exchange interaction connecting different rare-earth layers. The inverse of this expression characterizes the intrinsic time scale of the relative changes of the magnetization. This estimate suggests a characteristic time scale around a few ps, since the calculated exchange interactions are in the sub-meV range and the magnetic moments are of the order of $(1-10)\mu_{\mathrm{B}}$.

Further numerical simulations were performed with the simulation package UppASD[25] for a more precise statement on the rates. To have a perturbation from which the system evolves, the magnetic ground state was thermalized. Such a state is represented by the van Mises Fisher (vMF) distribution[26,27] of the width $2/\kappa$. In mean field approach, $\kappa = |\vec{B}|/k_B T_s$ is the ratio between the Weiss field $\vec{B}$ and (spin) temperature T_s. For going a beyond mean field interpretation, however, the connection between κ and the (spin) temperature T_s is resolved through Eq. (16) by Ma et al[28] (Fig. S3). The mean value of the magnetization of this distribution is zero. In this thermal state, we obtain the rate of angular momentum transfer between individual moments as $\mathrm{R} = \frac{1}{\mathrm{N}}\sum_{\mathrm{i}}|\partial \vec{m}_i/\partial t|$ and the projected Weiss-field as $B^{\mathrm{proj}} = N^{-1}\sum_i \vec{\mu}_i \cdot \vec{B}_i$, where N is the number of atoms in our simulation box and $\sum \vec{\mu}_i$ is the magnetic moment at zero temperature. The results of calculated value of R and B^{proj} are shown in Fig. S3 for $\kappa = 500$. It should be noticed that both the spin temperature T_s and the rates R typically increase with smaller κ, where the projected Weiss field decreases (not shown).

In general, we have demonstrated that this model can reproduce the linear trend of a growing angular momentum transfer rate shown in Fig 3a of the manuscript.

Lastly, with regard to Gilbert damping, Schoen et al[29] points out that it is directly proportional to the density of states at the Fermi level, and therefore does not directly scale with the de Gennes factor (see Fig. S3d), and therefore serves to simply reduce the rate R without affecting the linear trend. Indeed, the R values shown in Fig. S3b are up to an order of magnitude larger than experimental values, in which such channels (e.g. dissipation to the lattice) are expected.

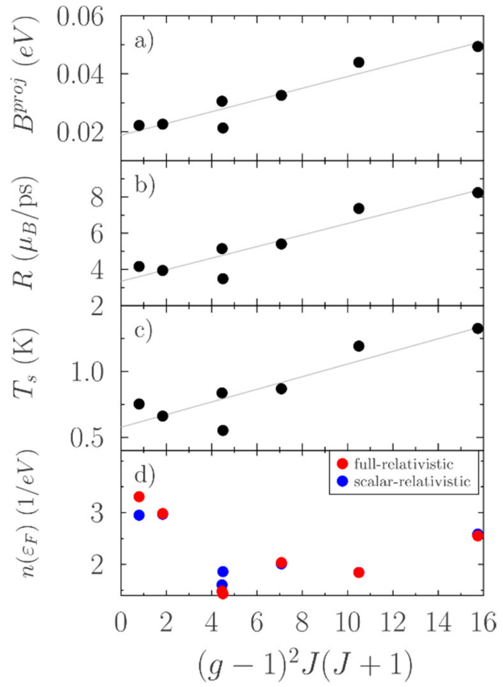

Fig. S3 – (a) projected Weiss field, (b) demagnetization rate, (c) spin temperature, and (d) DOS at the Fermi level, all shown as functions of the de Gennes factor for all the LnRh2Si2 compounds considered. The gray lines represent linear fits of the data. The vMF distribution width is $\kappa = 500$.

7. Calculated exchange couplings

The calculated values in Fig. 3b of the main text were calculated using the self-consistent Green's function method (described in the methods section), which is considered precise, but costly. The calculation was repeated with the RSPt method (see following), which is less costly and commonly employed. Here we detail the RSPt calculation, and then compare the results of the two methods.

The electronic properties of $LnRh_2Si_2$ compounds were calculated using density functional theory (DFT), as implemented in the all-electron full-potential fully relativistic electronic structure code RSPt[30–32] that uses linear muffin-tin orbitals as basis functions. The exchange-correlation energy in DFT is described using the PBE-parametrized[33] (*Perdew-Burke-Ernzerhof*) generalized-gradient approximation. Fermi smearing of the electronic occupations is used with temperature set to 1 mRy ≈ 158 K and the summation in the Brillouin zone is done using a (40x40x80) *k*-mesh shifted by half the grid step with respect to the Gamma point. Based on the calculated electronic structure, the atomistic magnetic interactions are determined using the magnetic force theorem within the approach proposed by Lichtenstein, Katsnelson, Antropov and Gubanov (LKAG approach)[34]. Magnetic interaction parameters j_{ij} are evaluated for the antiferromagnetic ground state, observed experimentally for most of the $Ln Rh_2Si_2$ systems, where the two rare-earth spins in the unit cell are antiparallel. Although RSPt is a fully relativistic code, the aforementioned calculations are performed in the non-relativistic limit where the spin-orbit coupling is not included. The obtained j_{ij} parameters correspond then to Heisenberg exchange and the considered system is mapped onto an isotropic Heisenberg model (see for example, Eq. (1) and the accompanying discussion by Borisov et al.[35]).

Fig. S4 presents three nearest-neighbor couplings as functions of de Genne's factor $G = (g-1)^2 J(J+1)$. The top row (a-c) presents Green's function method results, and the bottom row (d-f) presents RSPt results. In both cases the in-plane couplings, j_1 and j_2, exhibit non-monotonous variations, in contrast to the coupling between the planes (j_3), which exhibits a clear (linear) trend with respect to G. It should be noted that despite the sign changes of j_1 (RSPt) and of j_2 (Green's), the calculated magnetic ground state agrees with the measured antiferromagnetic order for all studied systems, due to the coupling between further atomic neighbors.

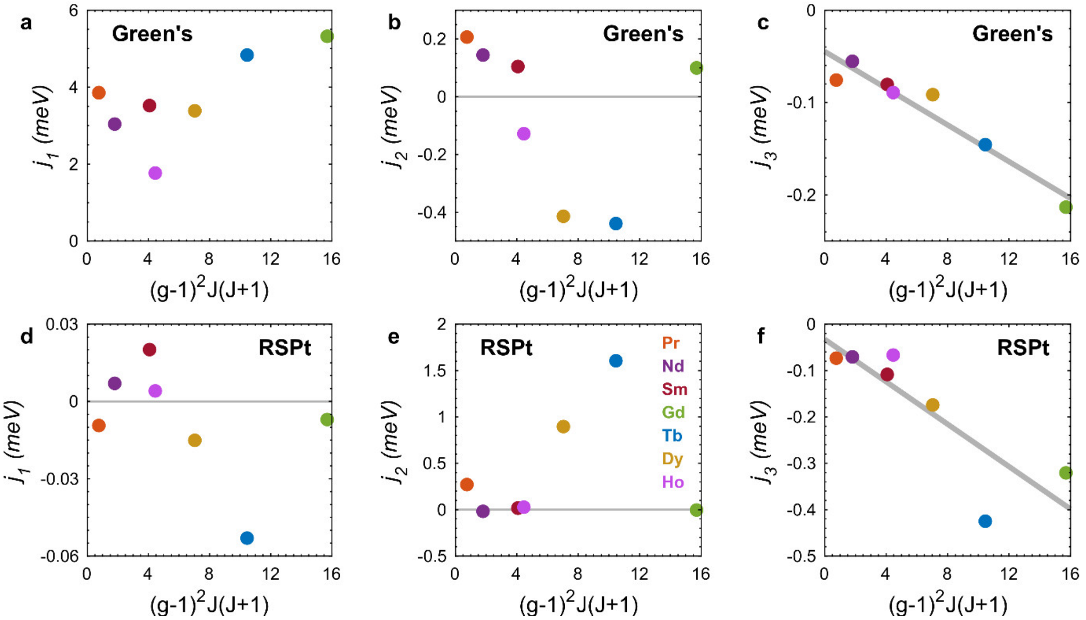

Fig. S4 – The three nearest neighbor couplings between *Ln* ions in $Ln Rh_2Si_2$ systems, as functions of de Genne's factor, calculated with two different methods: Green's function (a-c), and RSPt (d-f). The numbering of each coupling follows Fig. 3c of the main text.

8. The scalar-relativistic approximation in calculating electronic and magnetic structures

The electronic and magnetic properties of the *Ln*Rh_2Si_2 materials are distinguishable by the effect of the lanthanide elements within them, which harbor strongly localized 4*f* states. These 4*f* states form magnetic moments which interact primarily via free electrons, which in turn are characterized by the density of states at the Fermi level. The strong localization of the 4*f* electrons renders hybridization with valence states of other elements insignificant, so other types of magnetic interaction can be excluded. Thus, long range magnetic order in these compounds is driven primarily by the RKKY interaction.

The RKKY interaction can be represented mathematically by the size of 4*f* magnetic spins (not total moment) and the magnitude of the density of states (DOS) at the Fermi level. These properties can be calculated from first-principles if the localized nature of 4*f* elements is properly taken into account. To this end, in our work we used a GGA+U functional, in which the Hubbard parameter *U* was chosen such that it reproduces the experimental Neel temperatures. Although the Neel temperature is a macroscopic quantity, it can be evaluated using microscopic quantum mechanical calculations through the Heisenberg model. The exchange parameters in the Heisenberg model represent the overlap between various orbitals and can be obtained from first-principles calculations.

To reduce a substantial computational effort, our calculations of the exchange parameters were performed in a scalar-relativistic approximation, which neglects spin-orbit coupling. Although, several rare earth elements considered in our study (e.g. Dy, Sm, Ho) are characterized by a strong spin-orbit coupling, the scalar-relativistic approximation provides reliable results. To demonstrate the validity of this approach, we calculated the DOS of all materials considered in this study with both the scalar- and full-relativistic approaches (i.e. based on the Dirac equation without any further approximations). The comparison is shown in Fig. S5. Critically for our approximation, the value of the DOS at the Fermi level is reproduced in both calculations. Independently of the approximation employed, both approaches describe the compounds by a non-sharp DOS near the Fermi level and localized 4*f* electron states, and, therefore, are well fitted by a standard RKKY model[36].

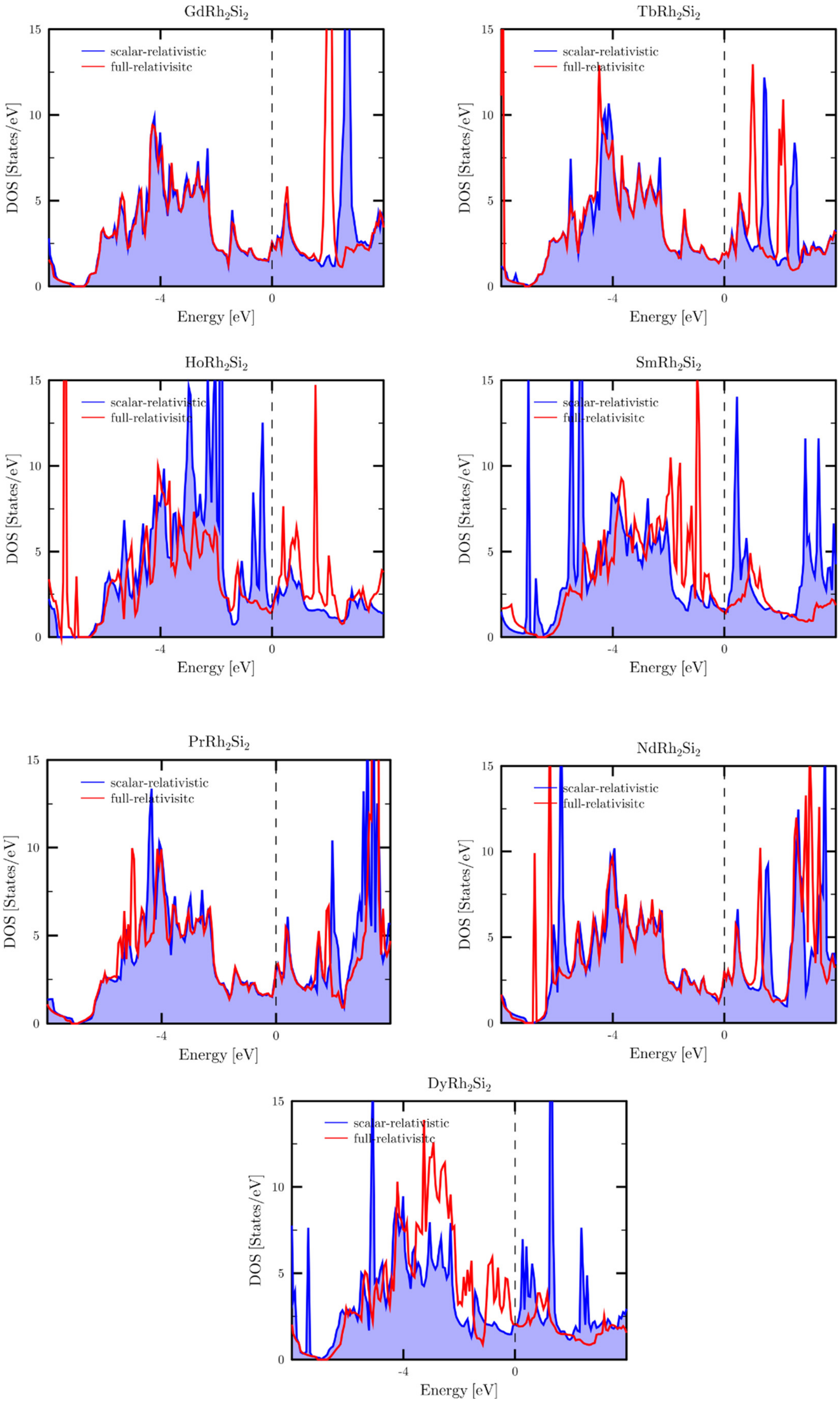

Fig. S5 – Comparison of calculated electronic density of states, as calculated by scalar-relativistic and full-relativistic approaches. Each panel presents calculations for a different compound considered in this study. Note the agreement of both calculations at the Fermi level.

9. Fits to demagnetization data

Here we present the best fits to demagnetization data used in this work:

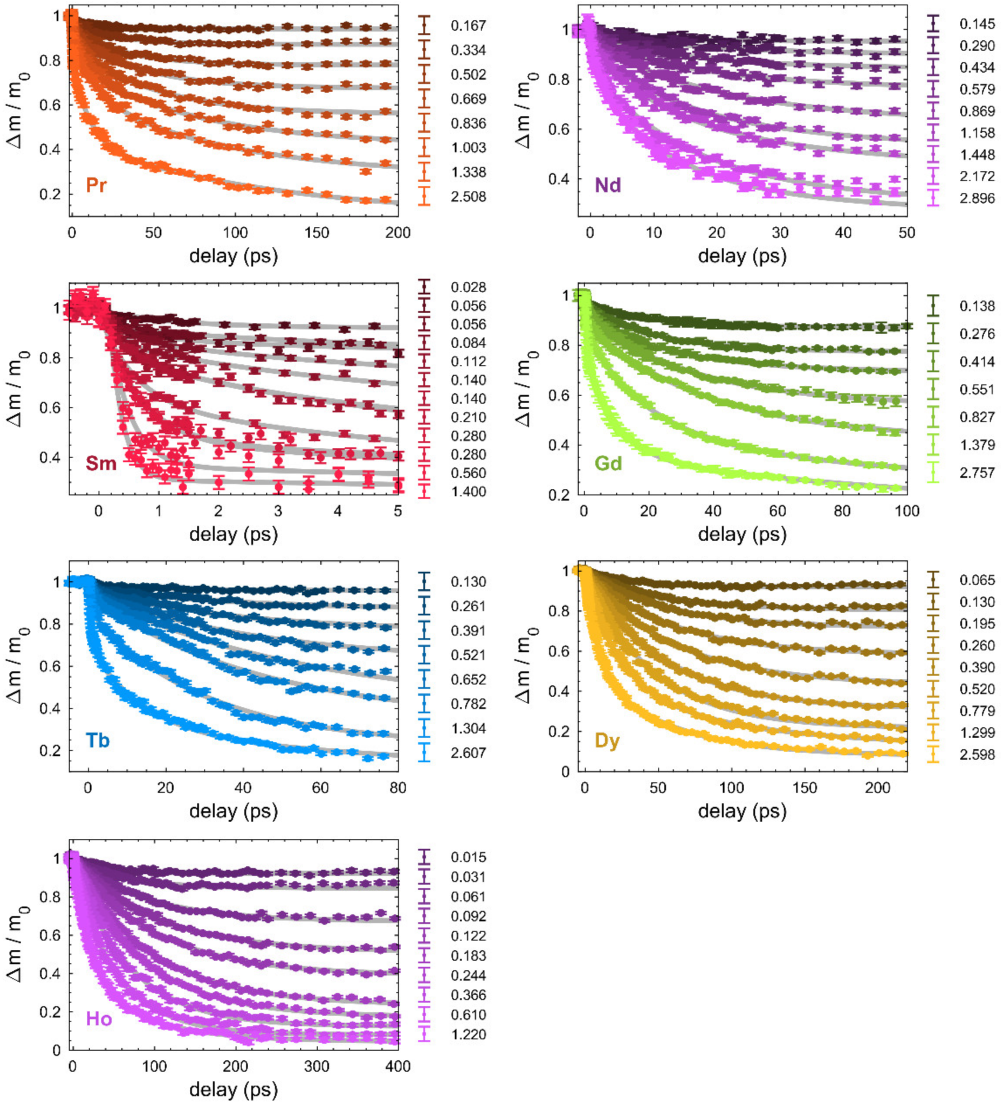

Fig. S6 – Demagnetization data used in this study. Lines are fits, as described in the Methods section of the main text. Legend values represent absorbed fluences.

10. Non-normalized parameters

Here we present the data shown in Fig. 2d,e of the main text, per-Ln ion, without normalizing the time scales by the values in the inset of Fig. 2e.

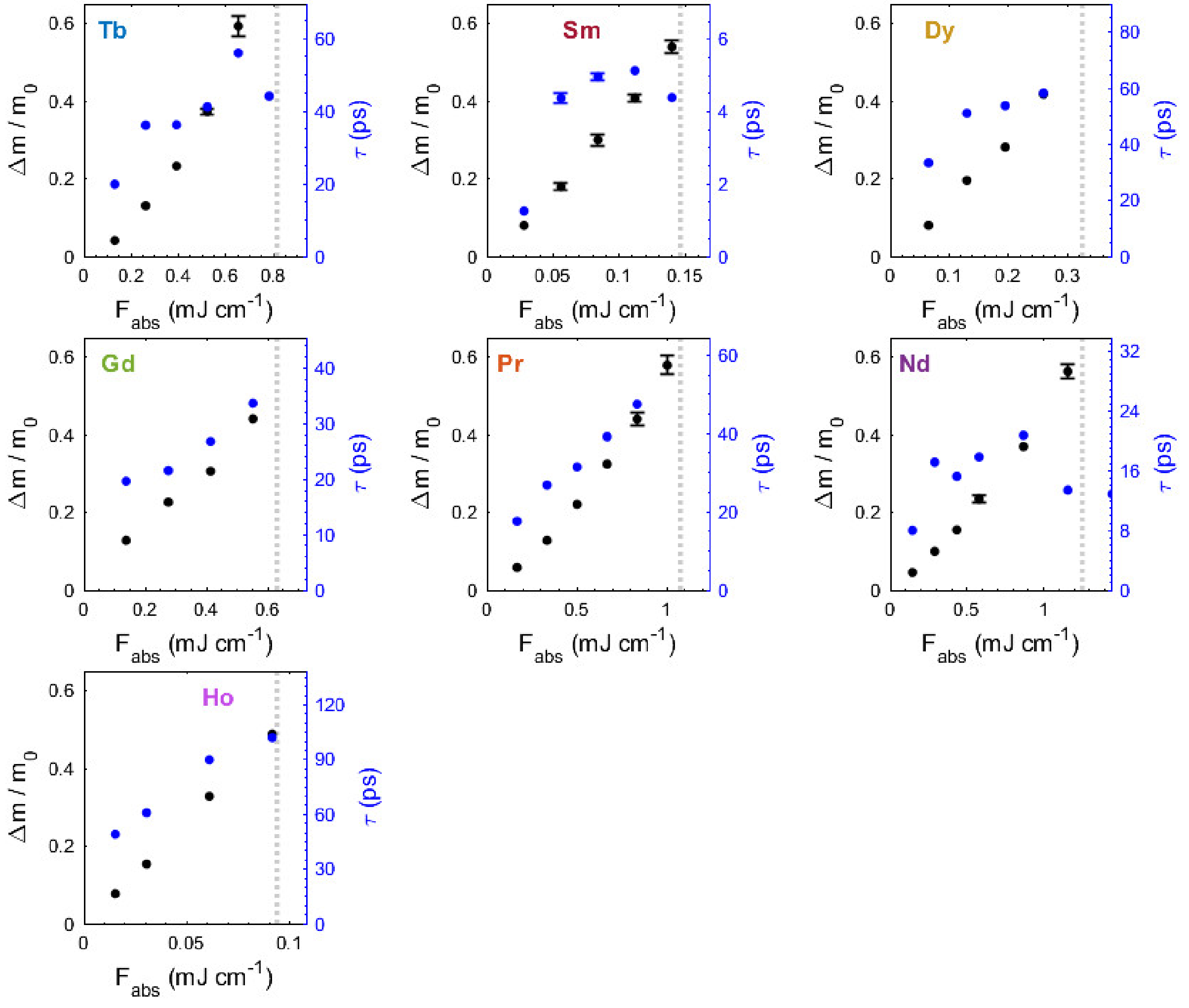

Fig. S7 – Fit parameters extracted from delay scans, presented per-element as functions of absorbed fluence, without normalizing the time scales. The data are the same as those presented in Fig. 2d,e of the main text. The dashed lines indicate the F_C values.

11. References

1. Fink, J., Schierle, E., Weschke, E. & Geck, J. Resonant elastic soft x-ray scattering. *Rep. Prog. Phys.* **76**, 056502 (2013).

2. Blume, M. Magnetic scattering of x rays (invited). *J. Appl. Phys.* **57**, 3615–3618 (1985).

3. Hannon, J. P., Trammell, G. T., Blume, M. & Gibbs, D. X-Ray Resonance Exchange Scattering. *Phys. Rev. Lett.* **61**, 1245–1248 (1988).

4. Ott, H., Schüßler-Langeheine, C., Schierle, E., Grigoriev, A. Y., Leiner, V., Zabel, H., Kaindl, G. & Weschke, E. Magnetic x-ray scattering at the M5 absorption edge of Ho. *Phys. Rev. B* **74**, 94412 (2006).

5. Hill, J. P. & McMorrow, D. F. Resonant Exchange Scattering: Polarization Dependence and Correlation Function. *Acta Crystallogr. Sect. A* **52**, 236–244 (1996).

6. Windsor, Y. W., Ernst, A., Kummer, K., Kliemt, K., Schüßler-Langeheine, C., Pontius, N., Staub, U., Chulkov, E. V, Krellner, C., Vyalikh, D. V & Rettig, L. Deterministic control of an antiferromagnetic spin arrangement using ultrafast optical excitation. *Commun. Phys.* **3**, 139 (2020).

7. Staub, U., Scagnoli, V., Bodenthin, Y., García-Fernández, M., Wetter, R., Mulders, a M., Grimmer, H. & Horisberger, M. Polarization analysis in soft X-ray diffraction to study magnetic and orbital ordering. *J. Synchrotron Radiat.* **15**, 469–476 (2008).

8. Flechsig, U., Nolting, F., Fraile Rodríguez, A., Krempaský, J., Quitmann, C., Schmidt, T., Spielmann, S. & Zimoch, D. Performance measurements at the SLS SIM beamline. *AIP Conf. Proc.* **1234**, 319–322 (2010).

9. Kachel, T., Eggenstein, F. & Follath, R. A soft X-ray plane-grating monochromator optimized~for elliptical dipole radiation from modern sources. *J. Synchrotron Radiat.* **22**, 1301–1305 (2015).

10. Holldack, K. *et al.* Femtospex: A versatile optical pump-soft x-ray probe facility with 100fs x-ray pulses of variable polarization. *J. Synchrotron Radiat.* **21**, 1090–1104 (2014).

11. Felner, I. & Nowik, I. Itinerant and local magnetism, superconductivity and mixed valency phenomena in RM2Si2, (R = rare earth, M = Rh, Ru)°. *J. Phys. Chem. Solids* **45**, 419–426 (1984).

12. Felner, I. & Nowik, I. Local and itinerant magnetism and superconductivity in R Rh2si2 (R = rare earth). *Solid State Commun.* **47**, 831–834 (1983).

13. Kliemt, K., Peters, M., Feldmann, F., Kraiker, A., Tran, D.-M., Rongstock, S., Hellwig, J., Witt, S., Bolte, M. & Krellner, C. Crystal Growth of Materials with the ThCr2Si2 Structure Type. *Cryst. Res. Technol.* **55**, 1900116 (2020).

14. Hossain, Z., Rajarajan, A. K., Anand, V. K., Geibel, C. & Yusuf, S. M. Magnetic properties of PrRh2Si2: A neutron diffraction study. *J. Magn. Magn. Mater.* **321**, 213–215 (2009).

15. Szytuła, A., Ślaski, M., Ptasiewicz-Bąk, H., Leciejewicz, J. & Zygmunt, A. Magnetic ordering in NdRh2Si2 and ErRH2Si2. *Solid State Commun.* **52**, 395–398 (1984).

16. Ślaski, M., Leciejewicz, J. & SzytuŁa, A. Magnetic ordering in HoRu2Si2, HoRh2Si2, TbRh2Si2 and TbIr2Si2 by neutron diffraction. *J. Magn. Magn. Mater.* **39**, 268–274 (1983).

17. Kliemt, K. & Krellner, C. Single crystal growth and characterization of GdRh2Si2. *J. Cryst. Growth* **419**, 37–41 (2015).

18. Kliemt, K., Banda, J., Geibel, C., Brando, M. & Krellner, C. Bulk properties of single crystals of the valence-unstable compound SmRh2Si2. *Mater. Res. Express* **6**, 126104 (2019).

19. Shigeoka, T., Fujiwara, T., Koyama, K., Watanabe, K. & Uwatoko, Y. High Field Metamagnetism of PrRh2Si2 Single Crystal Compound Having Anomalously High Nèel Temperature. *J. Low Temp. Phys.* **159**, 42–45 (2010).

20. Kampen, van, M. Ultrafast spin dynamics in ferromagnetic metals. (Technische Universiteit Eindhoven, 2003). doi:https://doi.org/10.6100/IR566741.

21. Koopmans, B., Malinowski, G., Dalla Longa, F., Steiauf, D., Fähnle, M., Roth, T., Cinchetti, M. & Aeschlimann, M. Explaining the paradoxical diversity of ultrafast laser-induced demagnetization. *Nat. Mater.* **9**, 259–265 (2010).

22. Atxitia, U., Chubykalo-Fesenko, O., Walowski, J., Mann, A. & Münzenberg, M. Evidence for thermal mechanisms in laser-induced femtosecond spin dynamics. *Phys. Rev. B* **81**, 174401 (2010).

23. Mendil, J., Nieves, P., Chubykalo-Fesenko, O., Walowski, J., Santos, T., Pisana, S. & Münzenberg, M. Resolving the role of femtosecond heated electrons in ultrafast spin dynamics. *Sci. Rep.* **4**, 3980 (2014).

24. Malik, R. S., Delczeg-Czirjak, E. K., Knut, R., Thonig, D., Vaskivskyi, I., Phuyal, D., Gupta, R., Jana, S., Stefanuik, R., Kvashnin, Y. O., Husain, S., Kumar, A., Svedlindh, P., Söderström, J., Eriksson, O. & Karis, O. Ultrafast magnetization dynamics in the half-metallic Heusler alloy Co2FeAl. *Phys. Rev. B* **104**, L100408 (2021).

25. Eriksson, O., Bergman, A., Bergqvist, L. & Hellsvik, J. *Atomistic Spin Dynamics: Foundations and Applications*. (Oxford University Press, 2017). doi:10.1093/oso/9780198788669.001.0001.

26. Watson, G. S. Distributions on the circle and sphere. *J. Appl. Probab.* **19**, 265–280 (1982).

27. Fisher, R. A. Dispersion on a sphere. *Proc. R. Soc. London. Ser. A. Math. Phys. Sci.* **217**, 295–305 (1953).

28. Ma, P.-W., Dudarev, S. L., Semenov, A. A. & Woo, C. H. Temperature for a dynamic spin ensemble. *Phys. Rev. E* **82**, 31111 (2010).

29. Schoen, M. A. W., Thonig, D., Schneider, M. L., Silva, T. J., Nembach, H. T., Eriksson, O., Karis, O. & Shaw, J. M. Ultra-low magnetic damping of a metallic ferromagnet. *Nat. Phys.* **12**, 839–842 (2016).

30. Wills, J. M. & Cooper, B. R. Synthesis of band and model Hamiltonian theory for hybridizing cerium systems. *Phys. Rev. B* **36**, 3809–3823 (1987).

31. Wills, J., Eriksson, O., Alouani, M. & Price, D. Full-Potential LMTO Total Energy and Force Calculations. in *Electronic structure and physical properties of solids* (Springer-Verlag, 2000).

32. Wills, J. M., Alouani, M., Andersson, P., Delin, A., Eriksson, O. & Grechnyev, O. *Full-Potential Electronic Structure Method, volume 167*. (Springer-Verlag, 2010).

33. Perdew, J. P., Burke, K. & Ernzerhof, M. Generalized Gradient Approximation Made Simple. *Phys. Rev. Lett.* **77**, 3865–3868 (1996).

34. Liechtenstein, A. I., Katsnelson, M. I., Antropov, V. P. & Gubanov, V. A. Local spin density functional approach to the theory of exchange interactions in ferromagnetic metals and alloys. *J. Magn. Magn. Mater.* **67**, 65–74 (1987).

35. Borisov, V., Kvashnin, Y. O., Ntallis, N., Thonig, D., Thunström, P., Pereiro, M., Bergman, A., Sjöqvist, E., Delin, A., Nordström, L. & Eriksson, O. Heisenberg and anisotropic exchange interactions in magnetic materials with correlated electronic structure and significant spin-orbit coupling. *Phys.*

Rev. B **103**, 174422 (2021).

36. Jensen, J. & Mackintosh, A. R. *Rare earth magnetism: structures and excitations*. (CLARENDON PRESS · OXFORD, 1991).
